# Responsible artificial intelligence for addressing equity in oral healthcare

**DOI:** 10.3389/froh.2024.1408867

**Published:** 2024-07-18

**Authors:** Zaid H. Khoury, Alexys Ferguson, Jeffery B. Price, Ahmed S. Sultan, Rong Wang

**Affiliations:** ^1^ Department of Oral Diagnostic Sciences and Research, School of Dentistry, Meharry Medical College, Nashville, TN, United States; ^2^ Department of Oncology and Diagnostic Sciences, University of Maryland School of Dentistry, Baltimore, MD, United States; ^3^ Division of Artificial Intelligence Research, University of Maryland School of Dentistry, Baltimore, MD, United States; ^4^ University of Maryland Marlene and Stewart Greenebaum Comprehensive Cancer Center, Baltimore, MD, United States; ^5^ Department of Oral and Craniofacial Sciences, School of Dentistry, University of Missouri-Kansas City, Kansas City, MO, United States

**Keywords:** artificial intelligence, interpretable models, explainable models, responsible models, equity, bias, oral healthcare

## Abstract

Oral diseases pose a significant burden on global healthcare. While many oral conditions are preventable and manageable through regular dental office visits, a substantial portion of the population faces obstacles in accessing essential and affordable quality oral healthcare. In this mini review, we describe the issue of inequity and bias in oral healthcare and discuss various strategies to address these challenges, with an emphasis on the application of artificial intelligence (AI). Recent advances in AI technologies have led to significant performance improvements in oral healthcare. AI also holds tremendous potential for advancing equity in oral healthcare, yet its application must be approached with caution to prevent the exacerbation of inequities. The “black box” approaches of some advanced AI models raise uncertainty about their operations and decision-making processes. To this end, we discuss the use of interpretable and explainable AI techniques in enhancing transparency and trustworthiness. Those techniques, aimed at augmenting rather than replacing oral health practitioners’ judgment and skills, have the potential to achieve personalized dental and oral care that is unbiased, equitable, and transparent. Overall, achieving equity in oral healthcare through the responsible use of AI requires collective efforts from all stakeholders involved in the design, implementation, regulation, and utilization of AI systems. We use the United States as an example due to its uniquely diverse population, making it an excellent model for our discussion. However, the general and responsible AI strategies suggested in this article can be applied to address equity in oral healthcare on a global level.

## Introduction

Oral health is defined as the condition of dental and orofacial structures that governs functions to be performed without pain, discomfort, or psychological distress (1). Maintaining oral health is essential to overall health and wellbeing (2). Oral diseases encompass a variety of conditions affecting the mouth, teeth, gums, and surrounding tissues. These diseases range from cavities (dental caries) and gum diseases (such as gingivitis and periodontitis) to more serious conditions like oral cancer. Many oral diseases are preventable and manageable through routine dental care and regular dental office visits. However, a significant portion of the population face barriers to accessing essential and affordable quality oral healthcare, rendering global oral health awareness and preventative care a formidable challenge (3). Statistically, dental caries is considered the most prevalent noncommunicable disease worldwide, affecting ∼2.5 billion people, whereas periodontal disease impacts ∼1 billion people (1). Furthermore, lip, oral, and pharyngeal cancers accounted for around 500,000 cases and 300,000 deaths in 2019, contributing to approximately 4.3 million disability-adjusted life years (DALYs) worldwide (4). Despite these alarming numbers, data on the distribution and sociocultural and behavioral determinants of oral health and disease across the globe are limited (5).

Addressing equity in oral healthcare is paramount, necessitating global efforts to reduce the public health burden of oral diseases through systematic analysis and research. The scope of oral health research is broad, encompassing epidemiology and public health research, community-based research, translational and clinical studies, behavioral and social sciences, infection control and microbiology, health disparities, and oral cancer research. Research in oral health has encountered numerous challenges related to accessibility, funding, methodology, and operation, impacting the development, creation, and implementation of effective oral healthcare policy and practice (6). Addressing equity involves implementing comprehensive strategies to ensure fair access, distribution of resources, and the reduction of disparities across diverse populations.

Artificial intelligence (AI) holds great potential in contributing to equity in oral healthcare but can also propagate bias and contribute to inequity if applied in a non-responsible manner. AI has been increasingly employed in diagnostic and therapeutic oral health applications (7) and has shown great promise in revolutionizing the field. However, the literature lacks clear insights on the potential role of AI in addressing equity in oral healthcare and research. Therefore, the aim of this mini review is to explore available interpretable and explainable AI models and examine their roles in promoting equity in oral healthcare and research while adhering to standards consistent with the responsible use of AI. We focus on the United States (U.S.) as an example due to its uniquely diverse population, making it an excellent model for our discussion. However, the general and responsible AI strategies suggested in this article can be applied to address equity in oral healthcare on a global level.

## The issue of inequity/bias in oral healthcare

Global inequities in oral health are the result of a multiplex of factors, some of which may be beyond an individual's direct control (1). These factors include issues related to affordability, accessibility, availability, and discrimination. Oral diseases are still considered a significant public health burden that affects low-socioeconomic and minority populations disproportionately despite ongoing research, increased oral health awareness, education, and widespread fluoride use (5, 8). In the U.S. for example, working adults with low incomes experience untreated dental caries two to three times more frequently than those with higher incomes, with financial constraints and lack of insurance coverage being the primary reasons for unmet oral healthcare needs (9). While oral health is an integral part of general health, there is variation across the globe regarding the inclusion of oral healthcare in public primary care institutions. In many regions, oral health services are treated as a separate entity affiliated with the private sector, thereby limiting the affordability of and accessibility to routine oral screening and treatment (10, 11).

The U.S. has the largest population of foreign-born residents in the world (12). People in the low-socioeconomic or uninsured category who are primarily from Black, Hispanic, Native, and other marginalized communities (13) are less likely to seek oral healthcare. This is evident from the fact that most of the documented oral health research focuses on White, non-Hispanic counterparts who can afford dental care and are more likely to have private dental insurance. Indeed, a greater portion of disease presentations affecting the oral cavity and the skin, particularly in textbooks, are skewed and focus mainly on White populations (14). Furthermore, residing in areas with social deprivation has been suggested to contribute to the higher caries risk observed among Hispanic and Black people (15), a cohort that experiences a higher tooth loss probability with age, as opposed to ageing White populations (16). The U.S. Census Bureau predicts that within 30 years, the non-white proportion of the American population will shift to more than 50% (17). A failure to create more racially diverse research cohorts could exacerbate existing health disparities if those most affected by disease continue to be excluded from research (17).

Furthermore, language barriers can hinder people's awareness, education, and access to oral healthcare. With approximately 7,168 languages spoken worldwide today, only 23 languages are spoken by the majority of the population (18). Particularly with increased worldwide travel and immigration, language barriers may delay oral healthcare due to misunderstanding of available services, operational times, cost of care, and operative instructions, highlighting the necessity for oral translation tools as part of the standard of care (19). Outreach populations, particularly those speaking an uncommon language, often face a shortage of oral healthcare coverage. Moreover, the majority of dental and oral health research publications are available only in English (20). Therefore, the incorporation of large language models such as ChatGPT during in-person or telehealth visits has the potential to reduce accessibility bias and in turn promote diversity in oral healthcare delivery, if implemented appropriately (21).

Bias in oral healthcare refers to any systematic preference, prejudice, or unfair treatment that may occur within the dental profession based on factors such as race, ethnicity, socioeconomic status, gender, sexual orientation, or other characteristics. Bias in oral healthcare fosters mistrust between minority patients and their dentists (22). In addition to racial and ethnic minorities, physically and mentally disabled individuals, persons living with HIV, and LGBTQ+ communities also fall under the minority category. Minority patients are often reluctant to visit the dentist due to feelings of shame, belittlement, judgment, or stress during the appointment. Such feelings may arise from reported negative past experiences, encountering poor chairside etiquette and differential treatment, and facing financial challenges which delay appropriate and timely treatment. As a result, the likelihood of returning to care or participating in oral research projects by this cohort is significantly reduced. This has a rippling effect, not only leaving a lasting negative impression on the patient, but also affecting the way minorities view other dentists and the profession which may influence the literacy and attitudes of their offsprings, family, and friends seeking oral healthcare. This could contribute to the lacking of participants from low-socioeconomic and minority populations in clinical studies and published data, which in turn hinders the progression towards unbiased oral health research (23).

## General strategies to address inequity/bias in oral healthcare & research

Making dental care available to vulnerable and underserved communities is not enough to rectify the lack of accessibility and oral health awareness within low-socioeconomic and minority groups. “Utilization”, “provision”, and “access” to services must all be addressed to achieve equity within oral healthcare and research (24). Specifically, access to services has three elements: financial affordability, physical accessibility, and acceptability (25). Governments, dental schools, and nongovernmental organizations worldwide have made great strides in improving oral healthcare availability, accessibility, provision, and awareness (1). In the U.S., Medicaid, a taxpayer-subsidized public health program aiming to help minority and low-income individuals afford health care, provides oral health benefits for eligible adults and children (26). Medicaid also provides a large multistate data set which allows for the evaluation of the various factors associated with early tooth loss (16). According to the Medicaid website, twenty-five states have developed their own state Oral Health Action Plan (OHAP) based on the Medicaid model, allowing them to determine the type of dental services covered. In fact, an increase in the utilization of pediatric preventive dental services is observed among Medicaid-enrolled children who are assessed through well-child visits (WCV) (27, 28). However, most states cover emergency dental services only and less than half include comprehensive oral healthcare coverage (26). To achieve comprehensive nationwide coverage, all states need to implement OHAPs that cover all types of oral care procedures. Furthermore, many private dental offices may not accept Medicaid due to factors such as the tedious application process, low reimbursement rates, reported social stigma in Medicaid participation, and the shortage of specialists within the Medicaid network (29). In addition, racial and ethnic disparities exist among dentists participating in Medicaid, with non-white dentists being more likely to treat Medicaid patients. Such providers often reside in rural and high-poverty areas, affiliated with large group practices and federally qualified health centers (30). According to infographics published by the American Dental Association Health Policy Institute (2020), most of the dental workforce in the U.S. are white dentists (∼70%), while Asian (18%), Hispanic (6%) and Black (4%) dental providers collectively constitute a minority, with a minimal increase in their proportions over the past two decades (31).

Dental schools are often seen as hidden gems within their communities because they are relatively accessible and provide dental services at a reduced cost regardless of the status of dental insurance. The World Dental Federation (FDI) has been encouraging dental schools to expand the scope of their outreach and community-based practices (32). Enhanced awareness among underserved communities about the services provided by nearby dental schools should be advocated. However, dental schools providing service to the public are also faced with challenges related to funding and patient retention (32, 33). To this end, it is important for service-providing dental schools to accommodate people with disabilities by facilitating accessibility to their facilities and comply with regulations established by national authorities, such as The Americans with Disabilities Act (ADA).

While the “utilization” of medical and dental services describes the use of services by individuals for preventive or curative care (25), the “provision” of services deals with the process of providing services according to the various available inputs (human resources, physical capital, and consumables) in the dental healthcare system (34, 35). Dentists in the commissioned Corps of the U.S. Public Health Service, the Indian Health Service and the National Health Service Corps all bear the responsibility of bringing greatly needed preventive and restorative oral healthcare and education to minority and low socioeconomic populations throughout the country. These federal services have long used student loan repayment incentives to successfully recruit dentists to work in underserved areas. Many of these dentists choose to locate permanently in those areas after fulfilling their contractual obligations to these agencies, providing long-term dental care to local patients (36).

Success in research participation, education and effective treatment comes from building relationships, trust and mutual understanding between providers and patients (37). Social media and internet platforms have enabled patients to explore and read reviews to find suitable dentists. Patients often choose dentists from the same cultural or ethnic background, fostering a sense of comfort and trust so that they would be encouraged to visit the dental office frequently and thus there is increased willingness to become a participant in future research projects. Moreover, having an office with friendly, multiracial staff and multilingual paperwork can help enhance patient satisfaction. When patients develop meaningful relationships with their dental care providers, they are more likely to refer others in the community to visit the office as well. This approach represents one strategy for enhancing the frequency of dental visits and prevention of caries and periodontal disease within minority and low socioeconomic communities.

## Responsible AI for addressing inequity/bias in oral healthcare

The responsible use of AI technologies by oral health stakeholders must feature at its core, planning considerations for the anticipation and management of AI-associated risks. To that end, a major resource is offered by the Artificial Intelligence Resource Center (AIRC) through the National Institute of Standards and Technology (NIST) (38). Particularly, the “AI Risk Management Framework” is intended to “improve the ability to incorporate trustworthiness considerations into the design, development, use, and evaluation of AI products, services, and systems”. Section 3 of the framework titled “AI Risks and Trustworthiness”, outlines guiding principles essential for developing transparent and responsible AI systems. Of specific importance is subsection 3.7 “Fair – with Harmful Bias Managed”, addressing concerns regarding equity and the mitigation of harmful bias and discrimination. The framework emphasizes that bias extends beyond data representativeness of underrepresented cohorts and encompasses systemic, computational, statistical, and human-cognitive biases. It is important to recognize and address these intrinsic biases during the training phase of AI models, as these biases may perpetuate harm to vulnerable individuals and exacerbate inequities if not addressed systematically at the outset of the AI developmental phase. The Artificial Intelligence Code of Conduct offered by the National Academy of Medicine is another point-of-reference framework that aims to better health care for all through ensuring the application of ethical and safe use of AI algorithms in healthcare (39).

Recent advances in AI technologies and its widespread adoption in healthcare have delivered significant performance improvements. However, these achievements often came with increased model complexity, turning them into “opaque black box” approaches (such as convolutional neural networks or CNNs) and raising uncertainty about their operations and decision-making processes. When deploying such AI systems in oral healthcare, a significant challenge faced by healthcare providers, model developers, and regulators is understanding how the prediction (output) is generated by a model and why the model makes such a prediction. The ambiguity and uncertainty of the “black box” AI systems pose significant obstacles for achieving trustworthiness, fairness, and equity in oral healthcare. Explainable Artificial Intelligence (XAI) is a rapidly evolving model that aims to address these concerns. Although there is no standard “textbook” definition for XAI (40), it generally refers to AI systems that provide explanations on why and how decisions are made, emphasizing efforts being made towards understandability, transparency, and trustworthiness in AI (40, 41). Explainability is a general term that encompasses *interpretability* in the AI domain and the two terms are often used interchangeably. XAI has gained more attention in the public domain, particularly in discussions around ethics, accountability, and transparency in AI systems, while *interpretable* AI has received more attention within the scientific community, where researchers actively explore techniques and methodologies to improve the interpretability of AI models. As advocated in the AI Risk Management Framework by the AIRC, “trustworthiness” must feature “explainable and interpretable” models in addition to models that are “accountable and transparent” (38). Therefore, both XAI and interpretable AI should be incorporated into AI systems for oral healthcare and research.

Technically, several XAI methods have been developed to demystify the “black box” nature of neural networks towards a more transparent and explainable architecture (42, 43). One of such methods is Class Activation Maps (CAMs) which visualize the regions of an image that contribute most to the predictions made by a CNN (44). For examples, two particular CAMS, the Gradient Weighted CAM (Grad-CAM) and High Resolution CAM (HR-CAM) have been shown to provide insights into the process of AI decision-making for oral cancer and other cancer types, with improved diagnostic performance and positive user feedback (45–47). Specifically, Grad-CAM generates heatmaps by computing the gradients of the target class score with respect to the feature maps of the last convolutional layers in the CNN while HR-CAM aggregates feature maps from several layers to create a high-resolution localization map (46). Other XAI methods such as Spectral Relevance Analysis (SpRAy) and Testing with Concept Activation Methods (TCAV) demonstrated promise in detecting visually distinctive noise in images with potential applicability on oral cancer (48). Specifically, SpRAy detects the presence of user-unknown strategies used in decision-making while TCAV relies on the user-provided samples of a concept to explain it (48). Integrating explainability into AI within oral healthcare will boost trust of healthcare providers and their patients, enhancing confidence in the institution (49). Those explainable and interpretable AI techniques, aimed at augmenting rather than replacing oral health practitioners' judgment and skills, have the potential to achieve personalized dental and oral care that is unbiased, equitable, and transparent (50).

Significant recent advances have been made towards the development and deployment of safe and trustworthy AI at a national level, with the U.S. government announcing the “First-Ever Consortium Dedicated to AI Safety” (51). This U.S. AI Safety Institute Consortium (AISIC) includes approximately 200 leading AI stakeholders from academia, industry, government, and civil society organizations and will be operated via NIST (52). Additionally, the NIH has developed a program to address the responsible use of AI in biomedical research called the NIH Common Fund's Bridge to Artificial Intelligence (Bridge2AI) program (53). Bridge2AI is unique in its ability to bring together healthcare and technical experts with social scientists and humanists. At its core, the initiative aims to create data sets that are ethically sourced, trustworthy, and accessible, by “developing software and standards to unify data attributes across multiple data sources and across data types” and through the creation of “automated tools to accelerate the creation of FAIR (Findable, Accessible, Interoperable, and Reusable) and ethically sourced data sets.” Those government AI initiatives will undoubtedly contribute to more responsible AI for enhanced trustworthiness, fairness, and equity in oral healthcare. Certainly, efforts that render XAI methods accessible will reduce any inadvertent health disparities associated with oral health technologies (54).

## Concluding remarks

Despite the surge in implementing AI in oral health over the past decade, ethical issues related to AI responsibility have been reported (55). Generally, a lack of universally adopted regulatory and ethical frameworks remains a significant challenge in ensuring responsible AI use in oral healthcare. Most AI oral healthcare applications are reported in computer sciences journals and are predominantly based on data generated from a select few countries (approximately seven countries). Additionally, the scarcity of studies sharing AI application codes hampers reproducibility and poses a barrier to addressing ethical issues (55).

The utilization of AI to address oral healthcare inequity is promising, yet it involves a comprehensive and multifaceted approach to ensure responsible implementation. This requires a global perspective integrating collaborative efforts across societal, infrastructural, and regulatory domains (56). Such efforts should aim to break down barriers between public and private healthcare sectors and various health disciplines and advocate the concept of “comprehensive patient care” on a global level. Hence it is anticipated that there will be an unprecedented impact of utilizing responsible AI in addressing inequity in oral healthcare and this can only be achieved by calling on all stakeholders involved in the design, implementation, regulation, and utilization of AI systems.
